# Identification of novel biomarkers for lupus nephritis

**DOI:** 10.17305/bb.2024.10450

**Published:** 2024-07-08

**Authors:** Zhengyue Liao, Liying He, Jiaojiao Fu, Xiaotong Zhou, Yong Li, Jing He, Yixin Liu, Jinlin Guo, Sijing Liu

**Affiliations:** 1 College of Medical Technology, Chengdu University of Traditional Chinese Medicine, Chengdu, China; 2 Chongqing Key Laboratory of Sichuan-Chongqing Co-construction for Diagnosis and Treatment of Infectious Diseases Integrated Traditional Chinese and Western Medicine, Chengdu, China; 3 State Key Laboratory of Southwestern Chinese Medicine Resources, College of Pharmacy, Chengdu University of Traditional Chinese Medicine, Chengdu, China

**Keywords:** Immune cells, lupus nephritis (LN), differentially expressed genes (DEGs)

## Abstract

Lupus nephritis (LN) is an autoimmune disease that rapidly progresses as a secondary consequence of systemic lupus erythematosus (SLE) and has a very poor prognosis. Therefore, this study aimed to identify characteristics of immune cell infiltration and investigate potential therapeutic targets using bioinformatics methods and the Murphy Roths Large/lymphoproliferation (MRL/*lpr*) mouse model. In this study, a total of 2810 differentially expressed genes (DEGs) were identified, which were primarily enriched in inflammatory and immune regulation pathways. From these DEGs, 226 immune-related genes (IRGs) were also identified. The single-sample gene set enrichment analysis (ssGSEA) revealed that patients with LN had increased infiltration of effector memory CD4^+^ T cells, effector memory CD8^+^ T cells, gamma delta T cells, myeloid-derived suppressor cells (MDSC), follicular helper T cells, Th1 cells, and Th2 cells, and this was closely correlated with the DEG–IRGs. Furthermore, the potential therapeutic biomarkers, CD244, S100 calcium-binding protein P (S100P), and vascular endothelial growth factor C (VEGFC), were identified by random forest approach (RFA), which were validated in LN mice. These findings provide new evidence and insights for further research on the diagnosis and treatment of LN by identifying critical genes and their associations with immune infiltration.

## Introduction

Systemic lupus erythematosus (SLE) is an autoimmune disease that affects various organs and systems in the body. It is characterized by the presence of immune complexes formed by antibodies produced by the body itself, which then bind to antigens. One of the affected organs can be the kidneys [1]. Among the organs affected, the kidneys are particularly vulnerable to damage due to their highly vascularized nature, leading to the development of lupus nephritis (LN). The clinical manifestations of LN resemble those of nephrotic syndrome or chronic glomerulonephritis, presenting with symptoms, such as edema, hematuria, proteinuria, hypertension, fever, and rash [2]. In recent years, the integration of gene sequencing technology and bioinformatics analysis has gained considerable popularity in identifying diagnostic and therapeutic biomarkers for diseases. The application of bioinformatics analysis enables the processing of large sample sizes and rapidly provides valuable insights into the disease. Consequently, several genes strongly associated with SLE have been discovered utilizing this approach. However, the application of bioinformatics analysis to study kidney tissue in LN is still limited [3–5]. This article aims to contribute to the identification of new biomarkers in LN, potentially paving the way for the development of effective therapeutic targets in the future.

The strong correlation between immune cell infiltration and therapeutic and clinical outcomes has been observed in various diseases, particularly autoimmune diseases. The immune system, which consists of the innate and adaptive systems, acts as the body’s primary defense against harmful microorganisms. The innate system, composed of phagocytes (neutrophils, macrophages), natural killer (NK) cells, basophils, and other cells, generates a quick response to invaders. In contrast, the adaptive system, which includes T cells and B cells, employs antigen-specific receptors. These receptors undergo multiple genetic rearrangements during development and establish an immune memory. NK cells possess characteristics of both innate and adaptive immune cells [6]. Considering the diverse cell types participating in the immune response, it becomes essential to assess the infiltration of immune cells. This evaluation aims to determine if variations in the composition of immune infiltrate can support the development of innovative immunotherapeutic agents that target these cells. Moreover, immune cell infiltration plays a crucial role in LN, as it involves the recruitment of immune cells into the renal tissue in order to produce cytokines and chemokines, which subsequently contribute to tissue damage [7]. However, our understanding of immune infiltration in LN remains incomplete. A previous study analyzed 22 immune cell infiltration features in LN using the CIBERSORT database, but it overlooked the significance of T helper cells and their associated core genes [8]. CD4^+^ T cells have been observed to significantly infiltrate the kidneys of LN patients [9], proliferating and differentiating into various subpopulations in response to antigenic stimulation. These distinct subpopulations comprise Th1, Th2, Th9, Th17, Th22, Tfh, and Treg cells [10], with a particular focus on maintaining the equilibrium between Th1–Th2 [11] and Th17–Treg balance [12]. The interaction, between Th17 and Treg cells plays a vital role in the progression of SLE. Individuals with SLE often exhibit reduced Treg cell count and impaired functionality, which directly links to increased disease severity in lupus patients [13]. Given the significance of T helper cells in LN, it is unreasonable to exclude them from the analysis of immune cell infiltration in LN kidneys. Moreover, most of the existing studies have only conducted bioinformatic analysis and have not been validated through experiments. Therefore, in this study, we developed a model for LN using key differentially expressed immune-related genes (IRGs) screened in a database. The infiltration of 28 immune cells, including Th1, Th2, and Th17, was then analyzed in LN patients compared to healthy individuals. Additionally, the relevance of candidate genes to LN was assessed using the Murphy Roths Large/lymphoproliferation (MRL/lpr) mouse model, and potential biomarkers were explored for the diagnosis and treatment of LN.

## Materials and methods

### Gene expression analysis

The RNA-seq data obtained from the Gene Expression Omnibus (GEO) series were utilized for the identification of differentially expressed genes (DEGs) between healthy individuals and patients with LN. In this study, three datasets (GSE112943, GSE157293, and GSE175759) were employed. The GSE112943 dataset contained 14 kidney samples derived from LN patients, while seven control kidney samples were also included. The GSE157293 dataset comprised three renal tissues from LN patients and three healthy renal tissues. The GSE175759 database included kidney samples from three cases of LN and 22 normal kidney samples. In order to minimize the influence caused by differences in technical and abiotic factors among these datasets, all of the data were re-normalized using the ComBat package and evaluated using principal component analysis (PCA). The DEGs between LN and control groups were filtered using the linear models for the microarray data (LIMMA) package, employing a cut-off value of Log2|fold change| > 1, and adjusted *P* < 0.05. Subsequently, volcano plots and heatmaps were used for visualization.

### Functional enrichment analyses of the identified differential genes

Protein interactions were analyzed, leveraging the search tool for recurring instances of neighboring genes (STRING) database. For the purpose of further exploring the connection between the DEGs, Gene Ontology (GO) and Kyoto Encyclopedia of Genes and Genomes (KEGG) enrichment analyses were conducted using the R software.

### Detection of IRGs

To acquire IRGs, the Immunology Database and Analysis Portal (IMMPORT) database (https://www.immport.org) was consulted, with a Venn analysis being carried out to ascertain the overlap genes between DEGs and IRGs. The intersection genes between them were considered as DE-IRGs.

### Immune infiltration analysis

To assess the distribution of 28 immune cell types within the kidneys of healthy individuals and LN patients, immune infiltration analysis was performed employing single-sample gene set enrichment analysis (ssGSEA). Moreover, the linear correlation between immune cells and DE-IRGs was assessed utilizing Pearson correlation analysis.

### Construction and assessment of diagnostic models

Random forest approach (RFA) was used to perform variable selection and determine IRGs for constructing LN monitoring models. Receiver operating characteristic (ROC) curves were generated based on the selected biomarkers from both the training and test sets. And the area under curve (AUC) was then calculated using the time ROC package in R software. The Gini coefficient, represented by the Gini index of inequality, was utilized as the partitioning criterion for the decision tree nodes in the random forest model. Based on the ranking of the Gini coefficients, the top 20 genes were selected for further analysis.

### Animals

Twenty female mice, ten with spontaneous SLE MRL/MPJ-Fas^lpr^ mice and ten control C57BL/6J mice, both at the age of eight weeks, were obtained from Nanjing R Born Biotechnology Co., Ltd., in Nanjing, China, under the animal certificate SCXK(Su)-2020-0009. The C57BL/6J mice are commonly used as a control for the MRL/MpJ-Fas^lpr^ mice [14, 15]. The mice were kept under specific pathogen-free (SPF) conditions at Chengdu University of Traditional Chinese Medicine. The room temperature was carefully maintained at 25 ± 2 ^∘^C, with a relative humidity of 65 ± 2%, and a 12-h cycle of light-darkness. After randomizing, the ordinary maintenance diet was provided to both the control group and the LN model group for a duration of nine consecutive weeks.

### Measurement of urine protein, creatinine, and albumin

To evaluate the protein and creatinine concentration in urine, urine samples were obtained using metabolic cages in nine weeks of administration at 24 h [16, 17]. The collected urine samples were centrifuged at 3000 *g*, for 5 min to remove all the particulates. After nine weeks of administration, we assessed the urinary protein level (Lot, C035-2-1), urinary creatinine level (Lot, C011-2-1), and urinary albumin level (Lot, C035-2-1) in mice. These measurements were conducted using commercially available kits provided by Nanjing Jiancheng Bioengineering Institute in Nanjing, China.

### Measurement of serum blood urea nitrogen (BUN), creatinine, and anti-dsDNA

Blood samples were collected from mice by orbital blood collection [18], and left to clot at room temperature for 1 h. The blood samples were then centrifuged at 3000 *g* for 10 min to collect the serum. Serum was acquired to ascertain indices, and the determination of the anti-double-stranded DNA (anti-ds DNA) antibody was performed using an ELISA kit obtained from Shanghai Enzyme-linked Biotechnology Co. (Lot, ml063514). Cobas C311 (Roche, Basel, Switzerland) was employed to determine serum BUN and creatinine levels, with BUN (Lot, 4460715190) and creatinine (Lot, 3263991190) kits from Roche.

### Hematoxylin–eosin (H&E) staining

Mice were anesthetized using 0.1 mL/10 g pentobarbital for sampling. The kidney tissue underwent paraffin embedding and was sectioned into 4 µm slices. Following this, the sections were stained with hematoxylin for a duration of 5 min. In order to dehydrate the sections, a gradient of 85% and 95% alcohol was used, each for 5 min. Afterward, the eosin stain was applied for another 5 min. Once the staining was completed, xylene was used to achieve transparency of the sections, which were then blocked using a neutral gel. Microscopic examination, image capture, and analysis revealed blue nuclei and red cytoplasm.

### Periodic acid-Schiff (PAS) staining

The paraffin-embedded kidney tissues were sliced into 4 µm sections. These sections were then dewaxed in water and treated with an iodic acid solution for 10 min. After this, Schiff’s reagent was applied for 30 min. The sections were then washed with running water and stained with hematoxylin for 3 min, followed by a 30-s rebluing with ammonia. A final round of rinsing under running water was performed before proceeding to the steps of dehydration, clearing, and sealing.

The staining was evaluated by two pathologists using a blinded method. The presence of glomerular congestion, leukocyte and monocyte infiltration, and tubular atrophy in the glomerular, tubular, interstitial, and vascular regions was evaluated based on methods outlined in previous literature [19]. The severity of these findings was graded as follows: 0 ═ 0%–5% staining; 1 ═ 5%–25% staining; 2 ═ 25%–50% staining; 3 ═ 50%–75% staining; 4 ≥ 75% staining.

### Quantification RT-PCR

Total kidney RNA was extracted using an RNA-easy isolation reagent (Lot, R701-01) purchased from Vazyme Biotech Co., Ltd., Nanjing, China. The reverse transcription process was conducted using the ExonScript RT SuperMix with dsDNase (Lot, A502-02, Exongen Biotech Co., Ltd., Chengdu, China). qRT-PCR reaction was carried out in a real-time PCR system (SLAN-96P, HONGSHI) using AceQ qPCR SYBR Green Master Mix (without ROX) (Lot, Q111-02, Vazyme Biotech Co., Ltd., Nanjing, China). Relative expression levels of target mRNAs were calculated using the 2^−ΔΔct^ method and then normalized. The primers used in qRT-PCR can be found in Table 1.

**Table 1:** Primer sequences

**Gene**	**Oligonucleotide sequence**	
*S100P*	Forward	5′-CCTCCCCTGACTTTGCCATT-3′
	Reverse	5′-GCAGTGCAGCTGAGATTTGG-3′
*CD209*	Forward	5′-CTGGCGTAGATCGACTGTGC-3′
	Reverse	5′-AGACTCCTTGCTCATGTCAATG-3′
*VEGFC*	Forward	5′-GAGGTCAAGGCTTTTGAAGGC-3′
	Reverse	5′-CTGTCCTGGTATTGAGGGTGG-3′
*SSTR1*	Forward	5′-CTACTGTCTGACTGTGCTTAGTG-3′
	Reverse	5′-CACGATGGGCAAGATAACCAG-3′
*CRLF3*	Forward	5′-AAGCTATTGGATGAGCGATTGG-3′
	Reverse	5′-TGACCCCATGCTCTATGAGTT-3′
*SDC1*	Forward	5′-CTTTGTCACGGCAGACACCTT-3′
	Reverse	5′-GACAGAGGTAAAAGCAGTCTCG-3
*CD244*	Forward	5′-CAGATGCTCAACTGTGGTTTCT-3′
	Reverse	5′-ACACTGTTCCGTTTCTGTAGGT-3′
*PTPRC*	Forward	5′-ACCACCAGGTGAATGTCAATTT-3′
	Reverse	5′-CTTGCTTTCCCTCGGTTCTTT-3′
*CD19*	Forward	5′-GGAGGCAATGTTGTGCTGC-3′
	Reverse	5′-ACAATCACTAGCAAGATGCCC-3′
*ADM*	Forward	5′-CACCCTGATGTTATTGGGTTCA-3′
	Reverse	5′-TTAGCGCCCACTTATTCCACT-3′
*JUN*	Forward	5′-CCTTCTACGACGATGCCCTC-3′
	Reverse	5′-GGTTCAAGGTCATGCTCTGTTT-3′

VEGFC: Vascular endothelial growth factor C; SSTR1: Somatostatin receptor 1; CRLF3: Cytokine receptor-like factor 3; SDC1: Syndecan 1; PTPRC: Protein tyrosine phosphatase receptor type C; ADM: Adrenomedullin; JUN: Jun proto-oncogene.

### Western blotting

Lysis of kidney tissue samples was conducted in the RIPA buffer containing protease and phosphatase inhibitors (Lot, AR0102S, Boster Biological Technology, Wuhan, China), followed by incubation on ice for a duration of 30 min. Centrifugation at 12,000 *g* for 10 min at 4 ^∘^C was then utilized to collect the supernatant through SIGMA 3-18KS. Subsequent quantification of protein levels in the supernatant was achieved via the employment of the BCA Protein Assay Kit, which was purchased from Mei5 Biotechnology, Co., Ltd. (MF071-01). The tissue lysate was separated to obtain total protein via the utilization of a 10% and 15% SDS-PAGE gel. Next, the separated protein was transferred onto a PVDF membrane. To block the membrane, a mixture of 5% skimmed milk in TBST buffer, which was dissolved in one sachet of TBS powder (Lot, T1086, Wuhan Solarbio Technology Co., Ltd.) in 1 L of purified water, adding 0.5 mL of tween 20, was applied at room temperature for 1 h. Later, incubation of the membrane was facilitated overnight at 4 ^∘^C with a diluted primary antibody. After undergoing a series of five washes with TBST, incubation with the corresponding secondary antibody (1:1000) was conducted at room temperature for 1 h. Following an additional five washes with TBST, chemiluminescence was initiated through the utilization of a developer solution and visualized in a gel imager. The immunoblot was then quantified using Image J software. For more detailed information on the primary and secondary antibodies, see Table 2.

**Table 2:** List of primary antibodies for western blotting

**Name**	**Host species**	**Reactivity**	**Cat. No.**	**Manufacture**
β-actin	Mouse	H, M	GB11001	Servicebio
S100P	Rabbit	H, M	P25815	Zenbio
VEGFC	Rabbit	H, M, R	251659	Zenbio
CD244	Rabbit	H, M, R	DF6741	Affinity
Multi-rAb HRP-Goat Anti-Mouse Recombinant Secondary Antibody (H+L)	Goat	M	RGAM001	Proteintech
Multi-rAb HRP-Goat Anti-Rabbit Recombinant Secondary Antibody (H+L)	Goat	R	RGAR001	Proteintech

VEGFC: Vascular endothelial growth factor C; H: Human; M: Mice; R: Rabbit.

### Immunofluorescence analysis

Kidney tissue sections were subjected to dewaxing in xylene, followed by dehydration utilizing a gradient ethanol series. Upon completion of antigen retrieval, incubation with 5% bovine serum albumin (BSA) (Lot, 4240GR025, German, BioFROXX) was conducted for a duration of 1 h at 92 ^∘^C. Subsequently, sections were stained with primary antibodies overnight at 4 ^∘^C. Incubation with a secondary antibody, was then performed, with final staining accomplished through utilization of DAPI (Lot, MBD0015, SIGMA). Stained sections were further subjected to dehydration, rendering them transparent, and visualized employing a microscope (FDR-6C, Cossim). For information about the primary and secondary antibodies utilized, refer to Table 2.

### Analysis of single-cell data

We collected sequencing data from kidney biopsies of 24 patients diagnosed with LN and ten healthy individuals, which were available publicly at www.immport.org/shared/study/SDY997. After merging the samples, we constructed single-cell analysis objects using the Seurat package. To ensure precise gene expression levels in the cells, we applied the “min. features” parameter. We also utilized the Uniform Manifold Approximation and Projection (UMAP) method for dimensionality reduction.

### Ethical statement

Approval for all experimental procedures conducted was granted by the Ethics Committee of the Affiliated Hospital of Chengdu University of Traditional Chinese Medicine, with the designated Approval No. 2022DL-019.

### Statistical analysis

The data were expressed as mean ± standard deviation (mean ± SD) and were analyzed using software version 9 of GraphPad Prism. To compare statistics, a *t*-test for independent samples was performed and adjusted with the Mann–Whitney rank sum test. Statistical significance was determined as *P* < 0.05.

## Results

### Characterization of differential mRNA in the kidney

**Figure 1.**
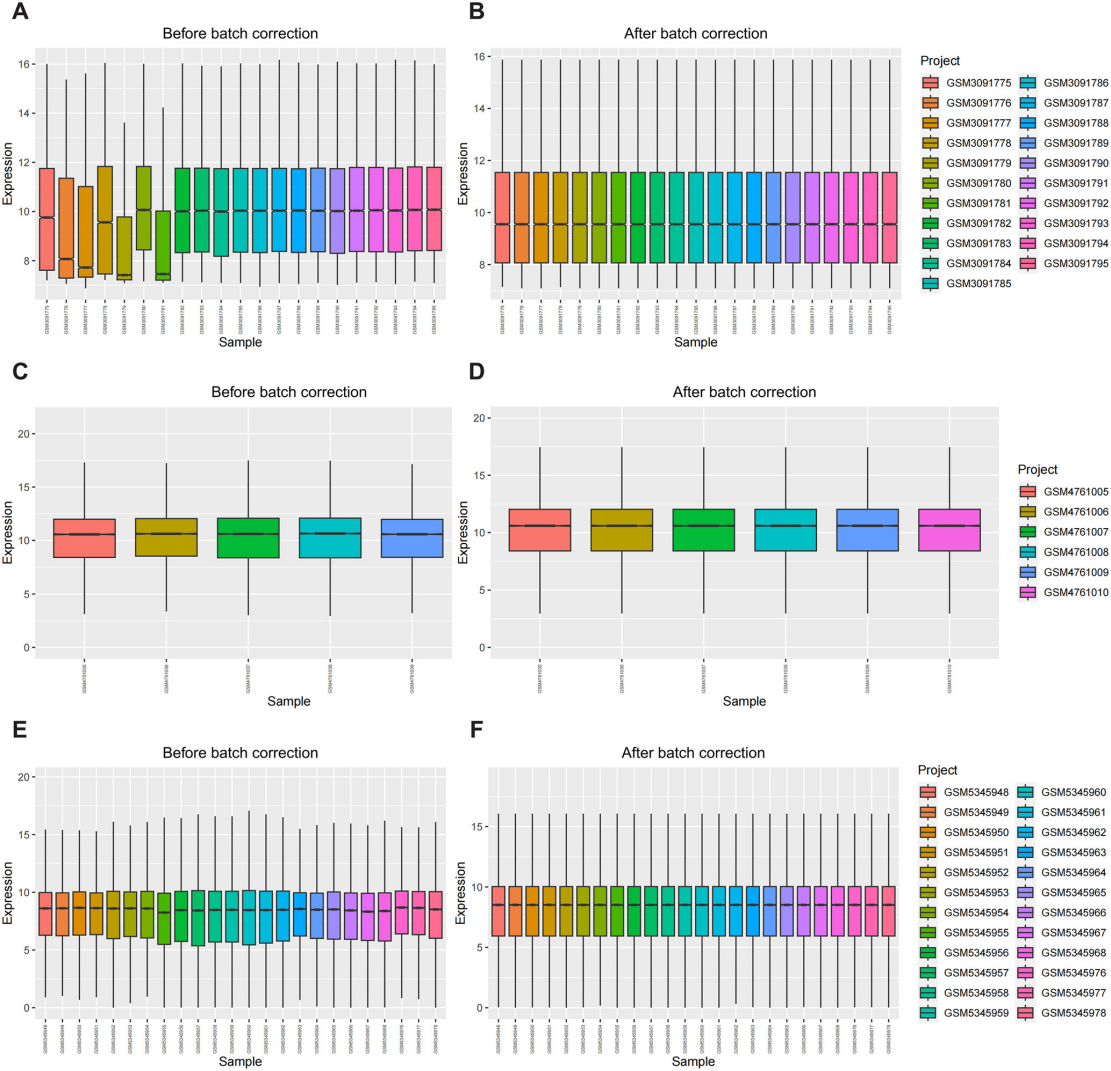
**Normalization of raw data.** Box plots of the expression levels of LN patients and control mRNAs before normalization (A, C, E) and after normalization (B, D, F) were plotted using the ComBat algorithm. The data were obtained from GSE112943, GSE157293, and GSE175759. LN: Lupus nephritis.

**Figure 2.**
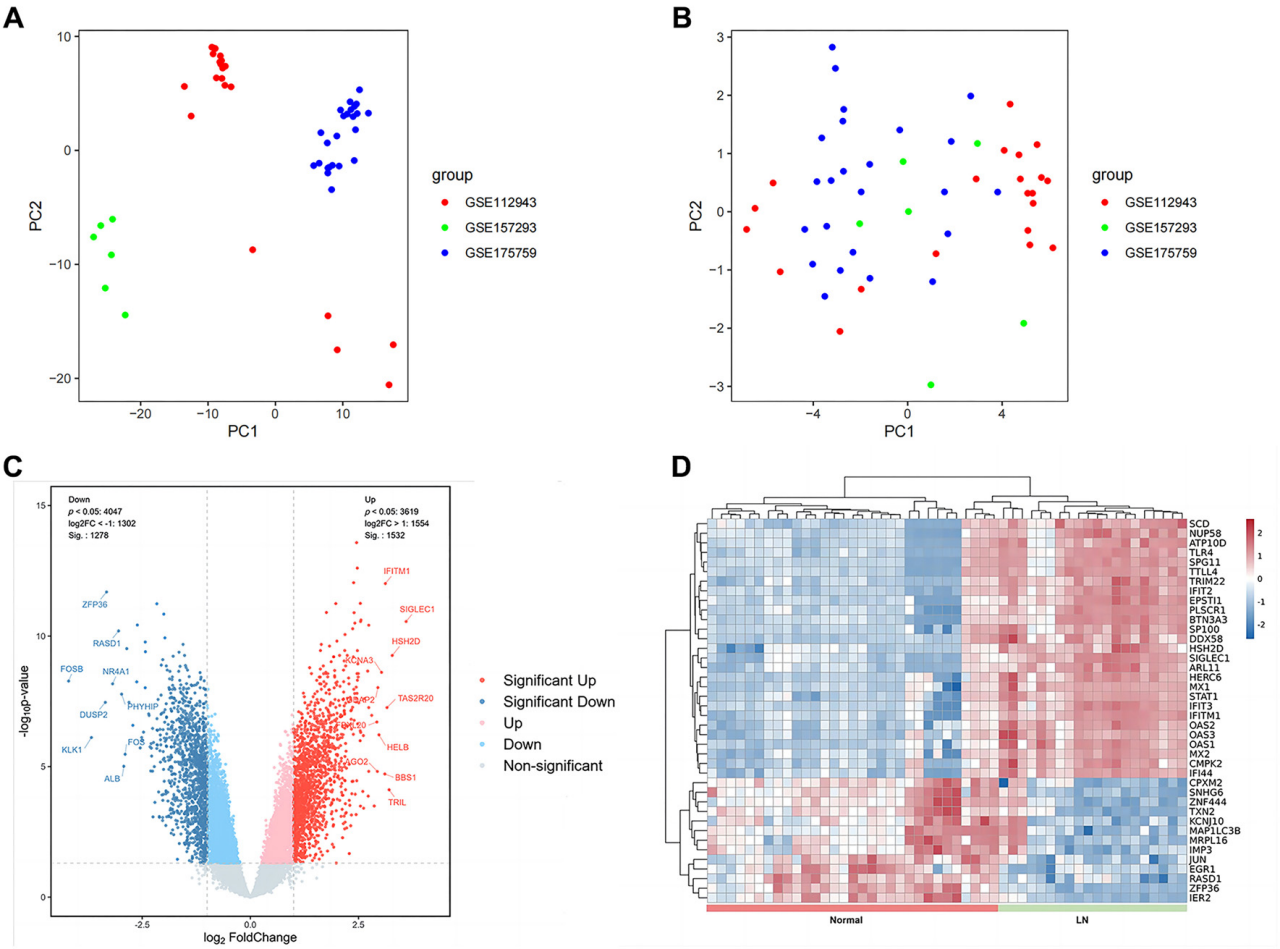
**Identification of DEGs.** (A) PCA clustering plot of GSE112943, GSE157293, and GSE175759 before batch effects were removed; (B) PCA clustering plot showing that the batch effects were removed; (C) Volcano plots of DEGs between LNs and controls; (D) Heatmap of LNs with the first 40 DEGs from healthy samples. Red: Upregulation; Blue: Downregulation; LN: Lupus nephritis; DEGs: Differentially expressed genes; PCA: Principal component analysis.

After removing the batch effect from three datasets related to LN (GSE112943, GSE157293, and GSE175759), we compiled a consolidated database consisting of gene expression profiles from 20 LN patients and 30 healthy individuals (Figure 1). PCA was conducted to validate the successful elimination of batch effects in the three datasets (Figure 2A and 2B). DEGs between the LN and control groups were then identified using the LIMMA package, with a cut-off value of Log2|fold change| > 1 and adjusted *P* < 0.05. A total of 2810 DEGs were identified across the three datasets, with 1532 upregulated genes and 1278 downregulated genes (Figure 2C). Among the upregulated genes in the LN group, the top five were *SIGLEC1*, *HSH2D*, *TRIL*, *TAS2R20*, and *IFITM1*, while the top five downregulated genes in the LN group were *FOSB*, *KLK1*, *DUSP2*, *ZFP36*, and *NR4A1* (Figure 2C). Additionally, there was variability in the expression of these DEGs between the LN group and the control group (Figure 2D).

### Functional enrichment analysis

**Figure 3.**
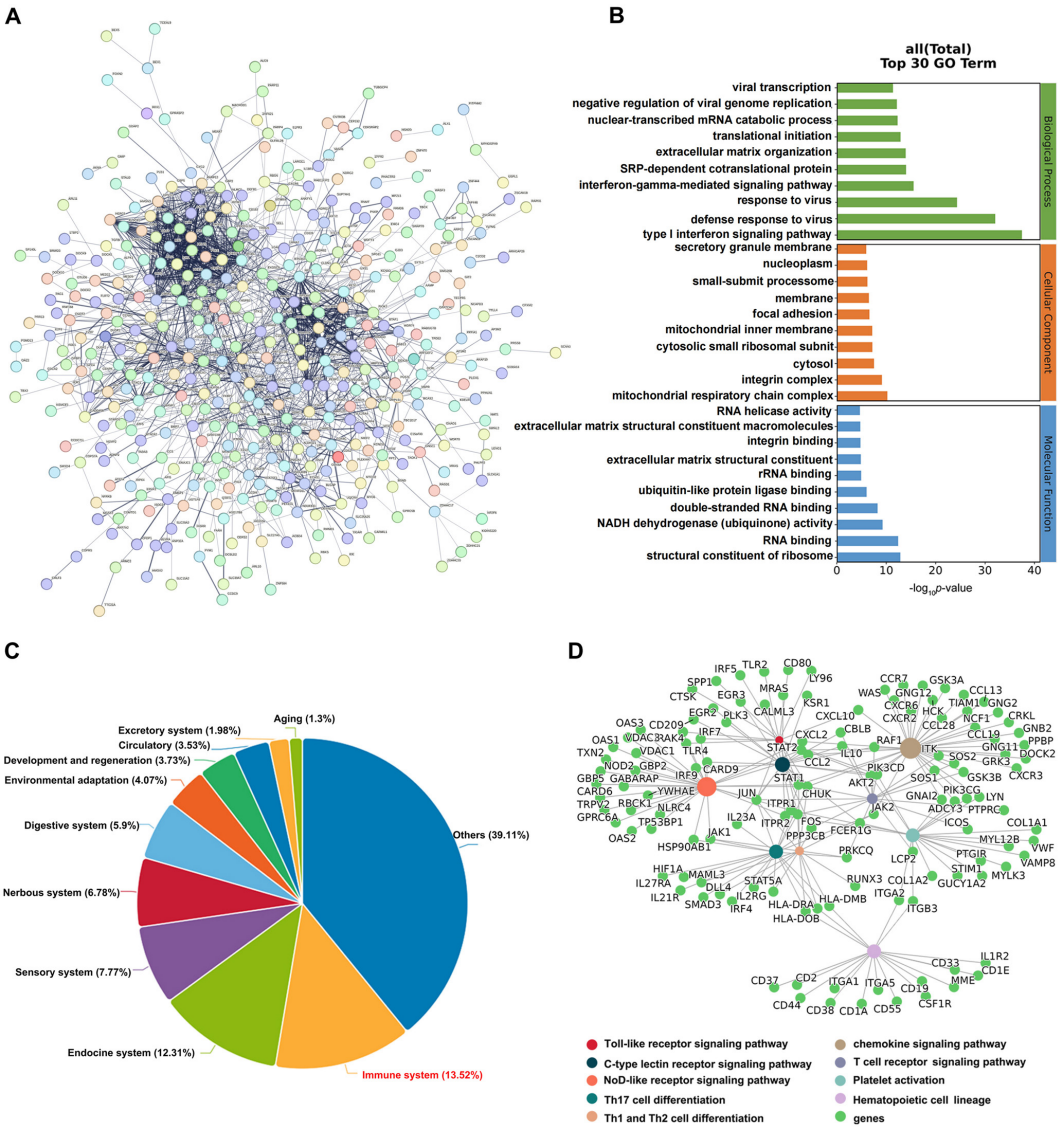
**Functional enrichment analysis.** (A) PPI network was constructed to reveal the interactions between different genes at the protein level; (B) GO analysis bar graph showed the enrichment of DEGs in biological process, cellular components, and molecular function processes in the enrichment of DEGs; (C and D) KEGG analysis plot shows that differential genes concentrate on the immune system, focusing on ten immune-related signal pathways. DEGs: Differentially expressed genes; GO: Gene Ontology; KEGG: Kyoto Encyclopedia of Genes and Genomes; PPI: Protein–protein interaction.

We constructed a protein–protein interaction (PPI) network to examine the interactions between DEGs, which revealed strong connectivity among the genes (Figure 3A). To further elucidate the functions and related pathways of DEGs, subsequent analysis for GO and KEGG enrichment was conducted. According to the findings, the DEGs participated in diverse biological processes, cellular components, and molecular functions. These encompassed the Type I interferon signaling pathway, nucleoplasm, and interactions with proteins in terms of binding (Figure 3B). Moreover, the KEGG enrichment analysis showed that the DEGs were predominantly enriched in the immune system (13.52%) (Figure 3C). Significantly, the major pathways of their dispersion included the differentiation of Th1 and Th2, Th17 cell differentiation, NOD-like receptor signaling pathway, Toll-like receptor signaling pathway, the signaling pathway of T cell receptor, C-type lectin receptor signaling pathway, chemokine signaling pathway, Fc gamma R-mediated phagocytosis, platelet activation, and the lineage of hematopoietic cells (Figure 3D).

### Immune infiltration analysis

**Figure 4.**
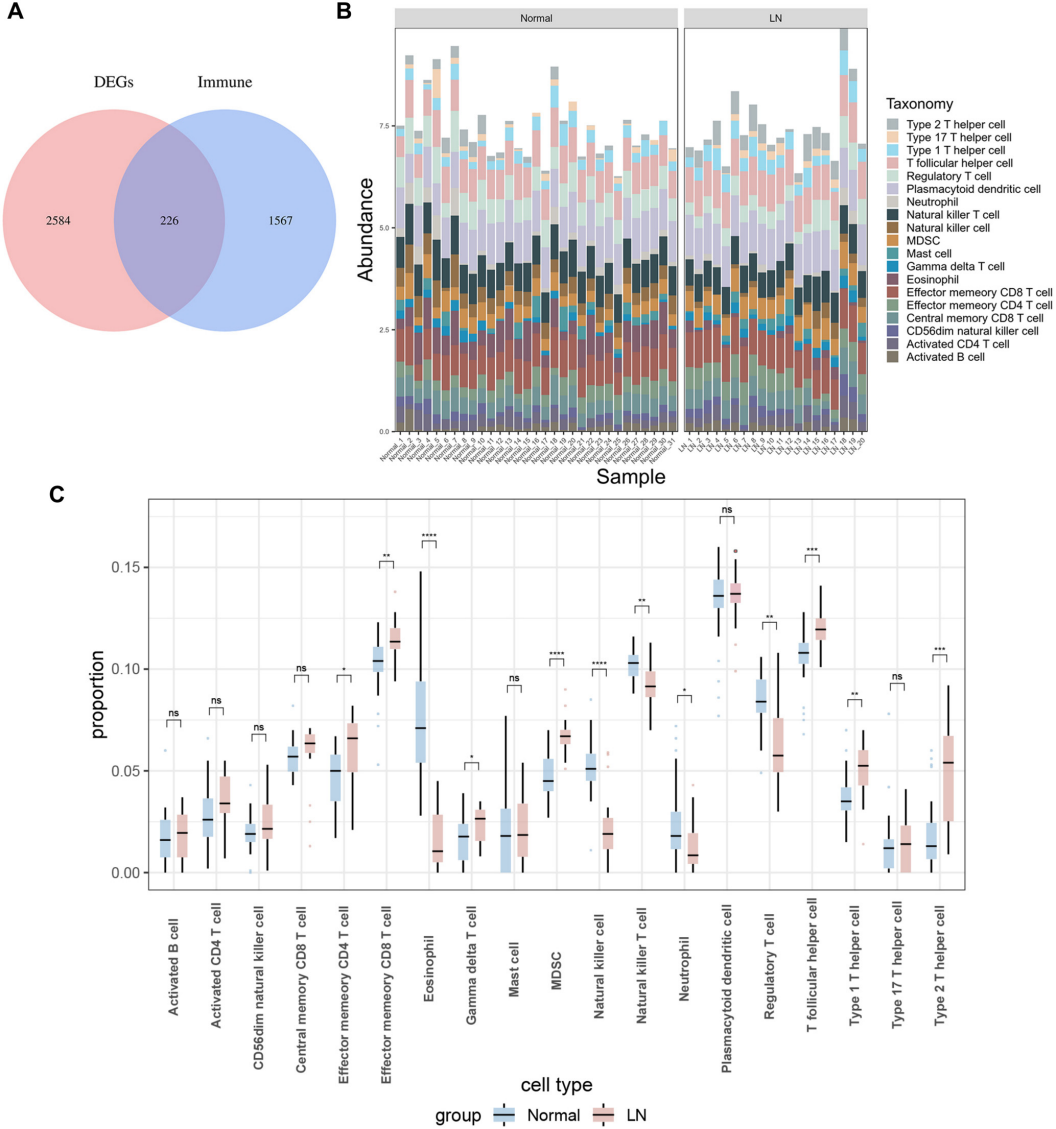
**Immune-related genes and corresponding immune cell subpopulations.** (A) Venn diagram of 226 DE-IRGs in LN; (B) Showing the changes of 28 major immune cells in LN; (C) Box plot of the difference in enrichment scores of the above immune cells between the LN group (red) and the control group (blue). *P value < 0.05, **P value < 0.01, ***P value < 0.001, ****P value < 0.0001. Ns: No significant; LN: Lupus nephritis.

To explore the IRGs in LN, we conducted a comprehensive analysis of a dataset comprising 2810 DEGs and 1793 genes associated with immunity. The findings, illustrated in Figure 4A, revealed the presence of 226 shared genes among them. We therefore investigated the relationship between these 226 genes and the infiltration of immune cells in specimens from LN patients and normal people. The immune infiltration analysis revealed significant differences in 12 immune cell subpopulations between the normal and LN groups. Specifically, patients with LN exhibited increased infiltration of effector memory CD4^+^ T cells, effector memory CD8^+^ T cells, Gamma delta T cells, myeloid-derived suppressor cells (MDSCs), follicular helper T cells, Th1 cells, and Th2 cells (*P* < 0.05). Conversely, a reduction in eosinophil, NK cell, neutrophil, and regulatory T cell infiltration was observed in patients with LN (*P* < 0.05) (Figure 4B and 4C).

### Determination of 20 key renal DE-IRGs in LN and construction of an immunodiagnostic model

**Figure 5.**
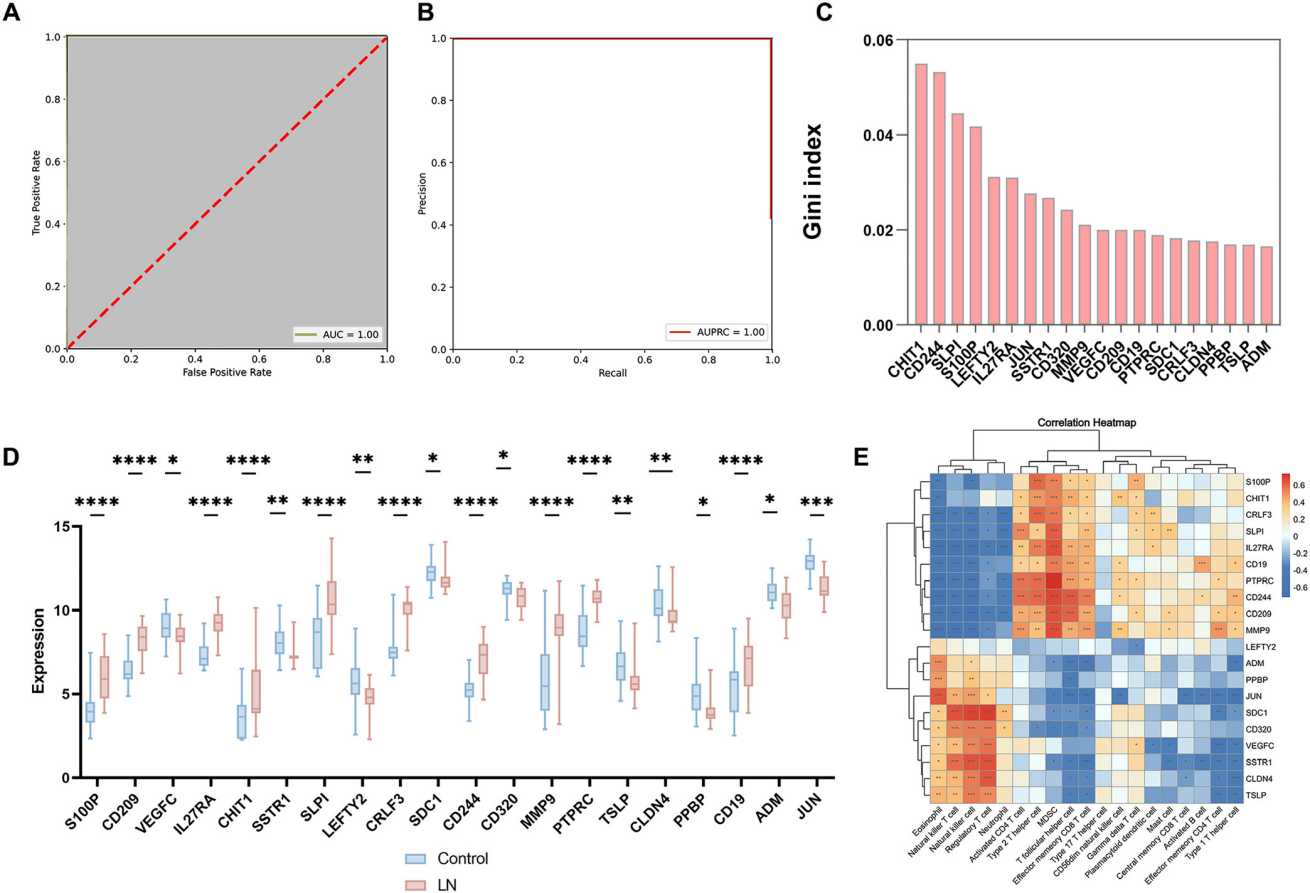
**Establishment and testing of gene diagnostic models.** (A and B) 20 DE - IRGs were identified as biomarkers by the RF method. ROC curves were evaluated for the diagnostic effects of the 20 genes; (C) The importance of the 20 selected genes was then assessed using the Gini index; (D and E) Heatmap plot showing the correlation of the 20 identified genes with 28 immune cells. Red: Upregulated; Blue: Downregulated. *P value < 0.05, **P value < 0.01, ***P value < 0.001, ****P value < 0.0001. IRGs: Immune-related genes; ROC: Receiver operating characteristic.

In order to further investigate the significance of key renal DE-IRGs in LN, the ranking of these genes based on their importance was determined through RFA. A set of 20 genes was collected through RFA to construct a diagnostic model for LN, and the efficiency of the identified genes was evaluated through an ROC curve, AUC ═ 1.00 (Figure 5A and 5B). The importance of the 20 selected genes was then assessed utilizing the Gini index, where a larger Gini index indicates greater gene importance (Figure 5C). Out of the 20 selected genes, S100 calcium-binding protein P (*S100P)*, CD209 Molecule (*CD209*), Interleukin-27 Receptor Subunit Alpha (*IL27RA*), Chitinase 1(*CHIT1*), Secretory Leukocyte Peptidase Inhibitor (*SLPI*), Cytokine Receptor-Like Factor 3 (*CRLF3*), CD244 Molecule (*CD244*), Matrix Metallopeptidase 9 (*MMP9*), Protein Tyrosine Phosphatase Receptor Type C (*PTPRC*), and CD19 Molecule (*CD19*) genes were upregulated (*P* < 0.05), while vascular endothelial growth factor C (*VEGFC)*, Pro-Platelet Basic Protein (*PPBP*), and Jun Proto-Oncogene (*JUN*) genes were downregulated (*P* < 0.05) (Figure 5D). In order to analyze the correlation between the expression of immune cells and DE-IRGs in LN, we conducted a study investigating the role of immune factors in the diagnosis and pathological mechanisms of LN. As illustrated in Figure 5E, we observed an increase in *S100P* expression in Type 2 T helper cells, MDSC, T follicular helper cells, effector memory CD8^+^ T cells, and gamma delta T cells, while a decrease was observed in eosinophils and NK cells.

### Verification of 20 differential genes between LN and control group in vivo

**Figure 6.**
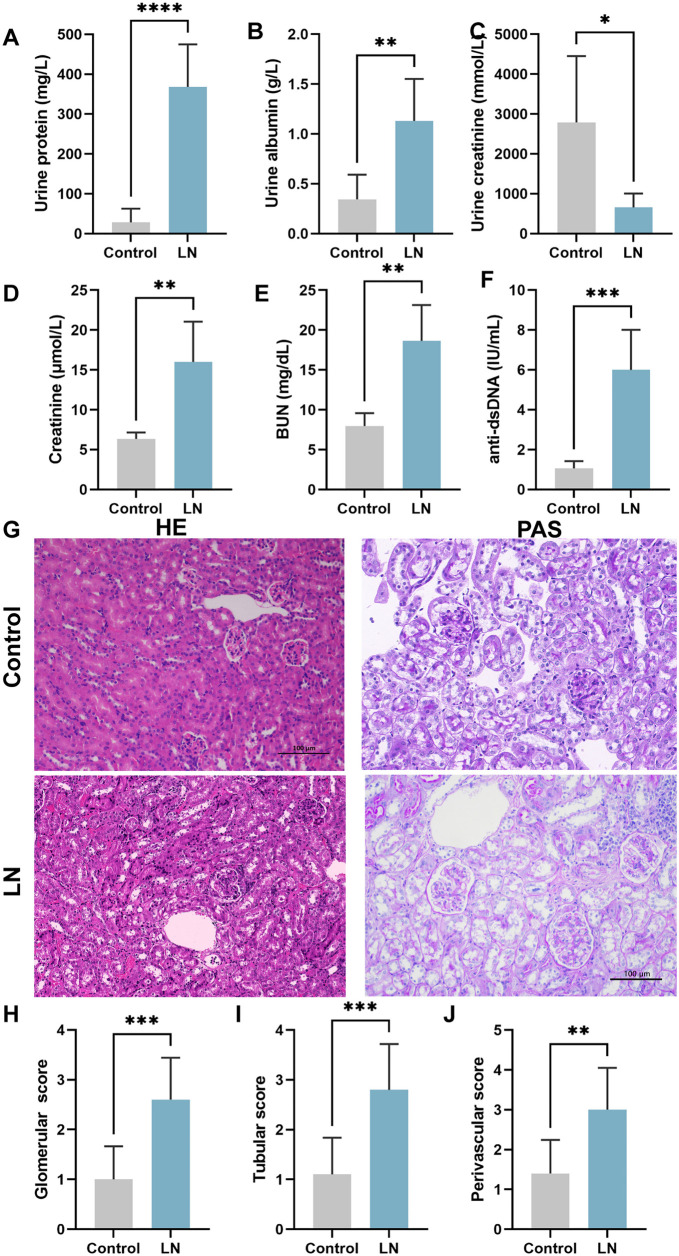
**Establishment of LN mouse model.** (A–C) Urinary protein and urinary albumin were significantly increased in the LN group compared to the control group, while urinary creatinine was significantly decreased; (D–F) Serum levels of creatinine, urea nitrogen, and anti-dsDNA were significantly elevated in the LN group of mice; (G) H&E staining and PAS staining; (H) Glomerular score; (I) Tubular score; (J) Perivascular score. 200 ×, bar ═ 100 µm. Black arrows indicate crescents, red arrows indicate lymphocytic infiltration. *P value < 0.05, **P value < 0.01, ***P value < 0.001, ****P value < 0.0001. *n* ═ 10 per group. LN: Lupus nephritis; PAS: Periodic acid-Schiff; H&E: Hematoxylin–eosin.

We confirmed our hypothesis by utilizing the MRL/MPJ-Fas^lpr^ mouse model of spontaneous LN, and the C57BL/6J mouse as the control group. In week 17 of our study, we observed a significant increase in urinary protein and albumin levels in the LN group, while urinary creatinine levels decreased (Figure 6A–6C). The LN group also showed elevated levels of serum creatinine, urea nitrogen, and anti-dsDNA (Figure 6D–6F). Notably, HE and PAS staining revealed glomerular swelling, increasing epithelial cell proliferation in the glomerular capsule wall layer, the accumulation of layers, the formation of crescent-shaped vesicles around the capillary plexus, renal tubular atrophy, and an elevated perivascular lymphocytic infiltration in the LN group in comparison to the control group. Additionally, there was an augmented proliferation of glomerular mesangial cells and collagen deposition in the LN group (Figure 6G–6J, *P* < 0.01). These findings provide strong evidence supporting the successful establishment of the LN mouse model.

**Figure 7.**
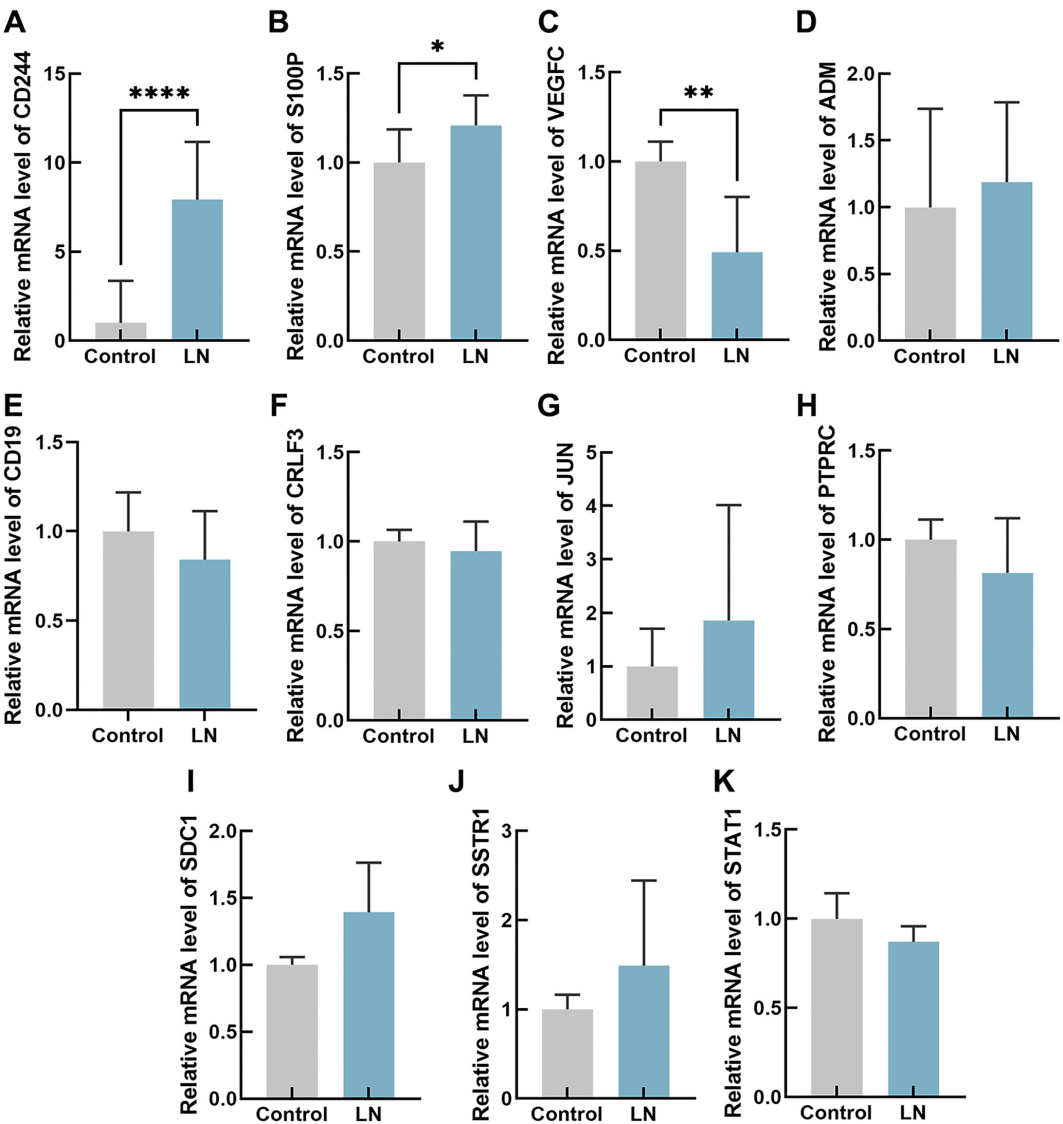
**Relative mRNA expression levels of CD244 (A), S100P (B), VEGFC (C), ADM (D), CD19 (E), CRLF3 (F), JUN (G), PTPRC (H), SDC1 (I), SSTR1 (J), and STAT1 (K) were determined by RT-qPCR.** *P value < 0.05, **P value < 0.01, ***P value < 0.001, ****P value < 0.0001. *n* ═ 10 per group. S100P: S100 calcium binding protein P; VEGFC: Vascular endothelial growth factor C; ADM: Adrenomedullin; CRLF3: Cytokine receptor-like factor 3; JUN: Jun proto-oncogene; PTPRC: Protein tyrosine phosphatase receptor type C; SDC1: Syndecan 1; SSTR1: Somatostatin receptor 1; STAT1: Signal transducer and activator of transcription 1.

**Figure 8.**
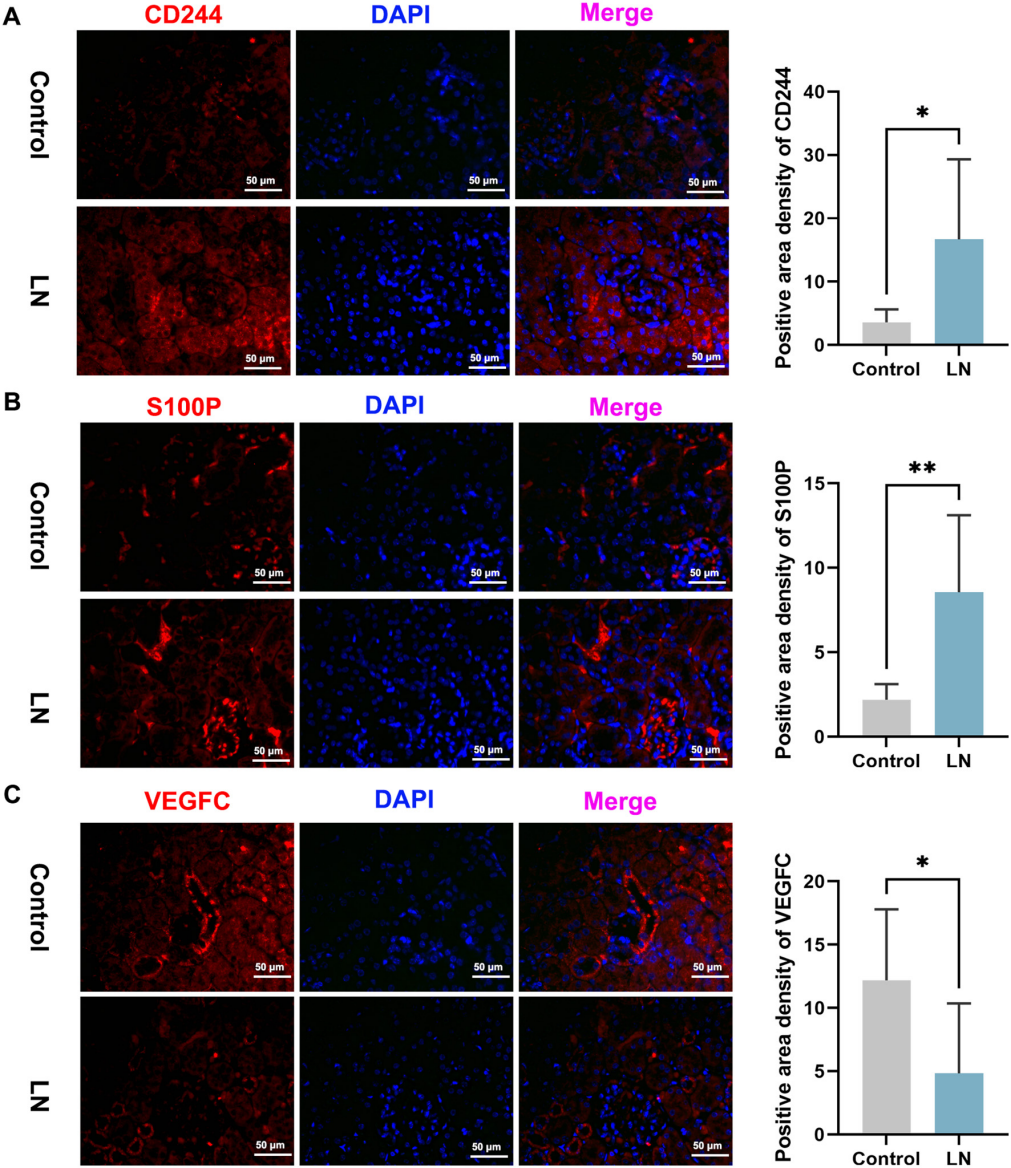
**Immunofluorescence reveals levels of CD244, S100P, and VEGFC in the kidney.** (A) CD244 (B) S100P (C) VEGFC. 200×, bar ═ 50 µm. *P value < 0.05, **P value < 0.01, ***P value < 0.001. *n* ═ 5 per group. S100P: S100 calcium binding protein P; VEGFC: Vascular endothelial growth factor C.

**Figure 9.**
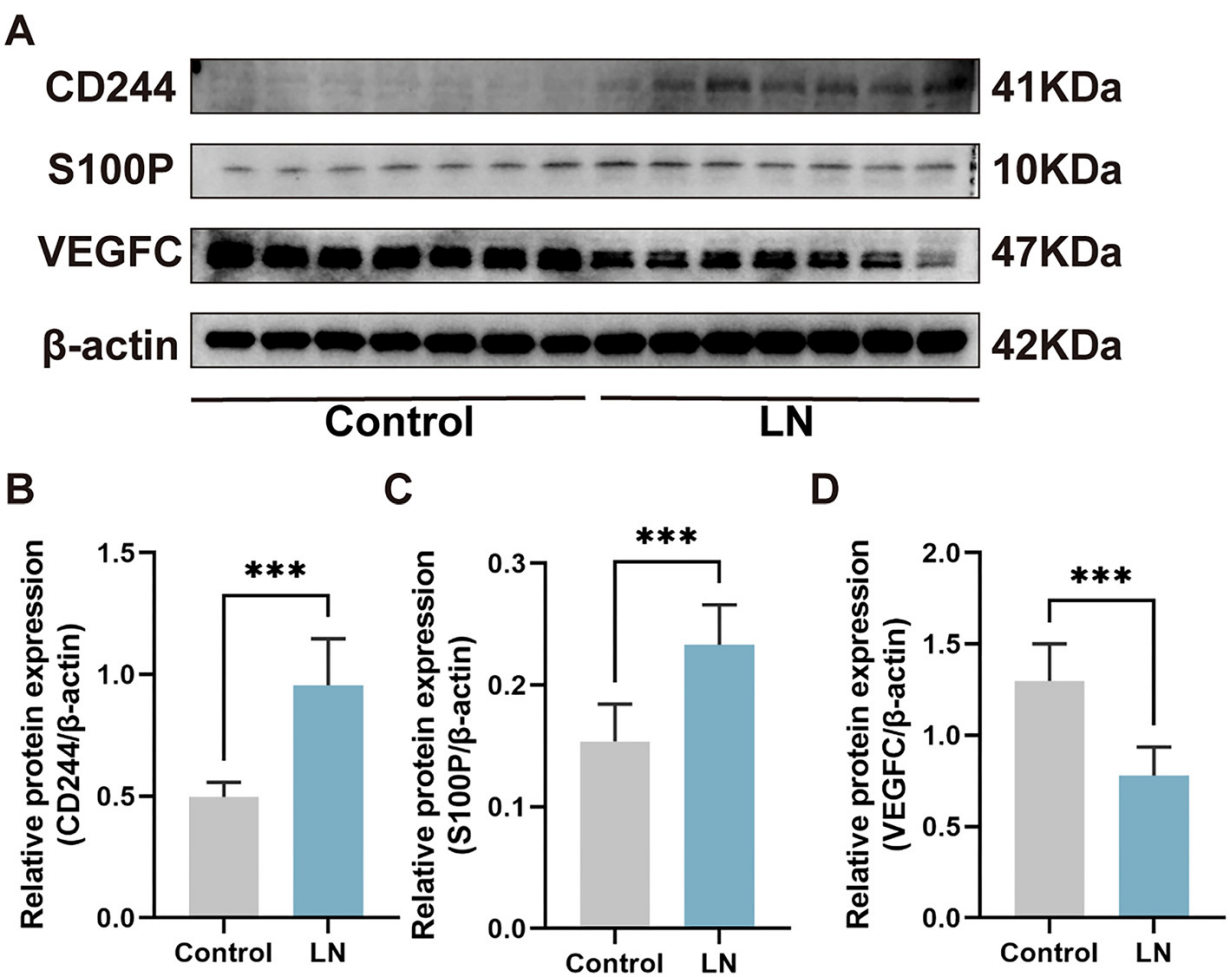
**Western Blotting reveals levels of CD244, S100P and VEGFC in the kidney.** (A) Protein level of CD244, S100P and VEGFC in the kidney; (B) Gray scale analysis of S100P; (C) Gray scale analysis of VEGFC; (D) Gray scale analysis of CD244. ***P value < 0.001. *n* ═ 7 per group. S100P: S100 calcium binding protein P; VEGFC: Vascular endothelial growth factor C.

In order to authenticate our findings, mouse kidney total mRNA was extracted and analyzed. The results indicated that in the LN group, *CD244* exhibited significantly higher levels compared to the control group (*P* < 0.0001) (Figure 7A), and *S100P* level was increased in the LN group (*P* < 0.05) (Figure 7B), while *VEGFC* was significantly decreased in the LN group (*P* < 0.01) (Figure 7C), which correspond to the preceding results (Figure 5C). However, there were no significant differences in the expression levels of Adrenomedullin (*ADM*), *CD19*, *CRLF3*, *JUN*, *PTPRC*, Syndecan 1 (*SDC1*), Somatostatin Receptor 1 (*SSTR1*), and signal transducer and activator of transcription 1 (*STAT1*) between the two groups (*P* > 0.05) (Figure 7D–7K). To verify the expression of *CD244*, *VEGFC*, and *S100P* in the kidney, western blotting and immunofluorescence assays were conducted. The study uncovered that CD244 and S100P exhibited notably elevated levels in the kidneys of LN mice in comparison to the control group. Furthermore, there was a decrease in the expression of VEGFC in the LN mice (Figures 8 and 9). These results suggest that CD244, S100P, and VEGFC may serve as potential biomarkers for LN.

### Distribution of *CD244*, *S100P*, and *VEGFC* in cells

**Figure 10.**
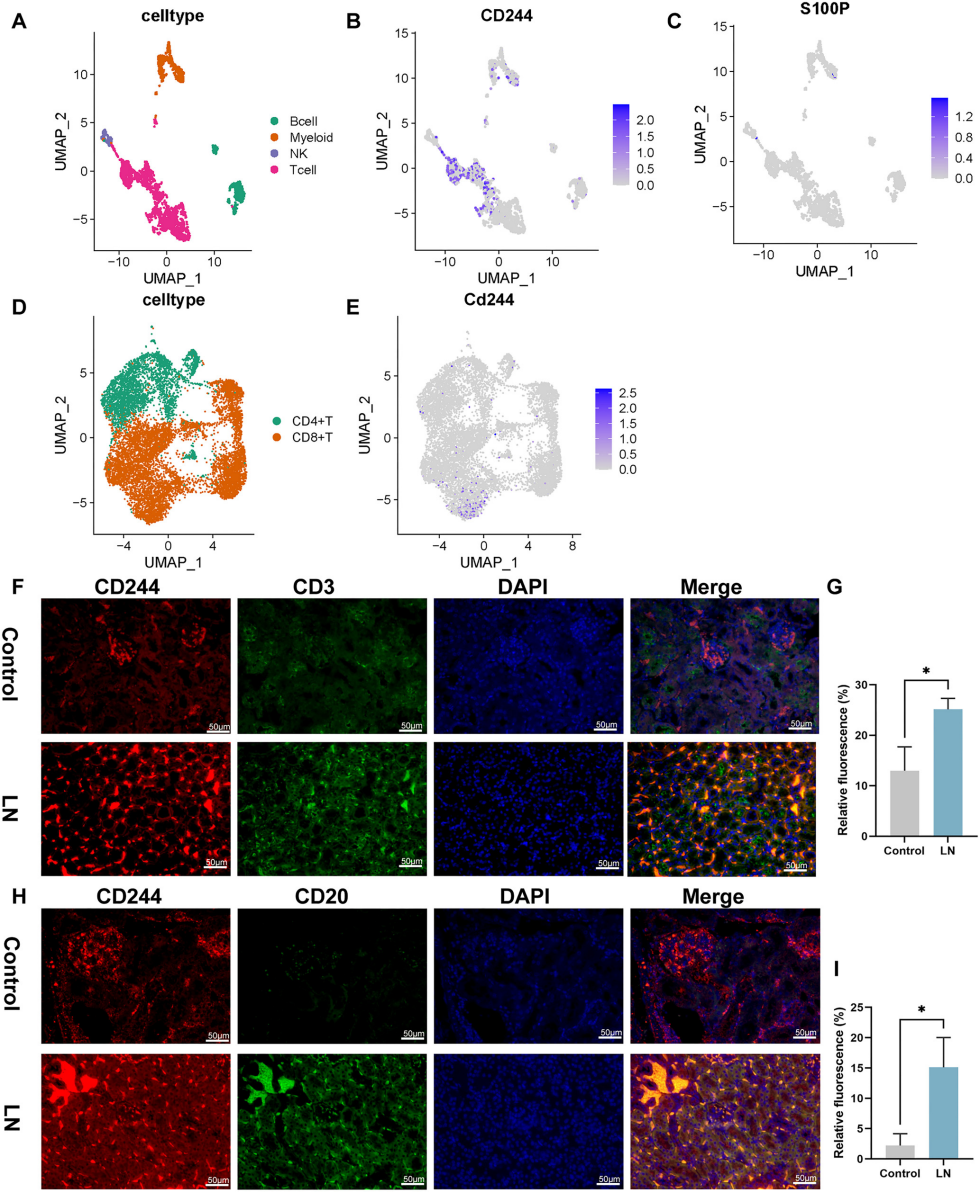
**Single-cell sequencing and immunofluorescence validation.** (A) The distribution of T cells, B cells, NK cells, and monocyte macrophages in the kidney; (B) Distribution of CD244 in each cell population; (C) The distribution of S100P in each cell population; (D) The distribution of CD4^+^ T cells and CD8^+^ T cells in the kidney; (E) The distribution of CD244 in the cell populations; (F–G) Relative fluorescence of CD244^+^CD3^+^; (H–I) Relative fluorescence of CD244^+^CD20^+^. 200 ×, bar ═ 50 µm. *P value < 0.05, **P value < 0.01, ***P value < 0.001. *n* ═ 5 per group. S100P: S100 calcium binding protein P; NK: Natural killer.

The distribution of *CD244*, *S100P*, and *VEGFC* in different cell populations of the kidneys of LN patients was analyzed using single-cell sequencing data from the IMMOPRT database. Spatial dimensionality reduction clustering was performed using the UMAP algorithm. The analysis revealed that CD244 was found to be distributed in myeloid, B cells, and NK cells, with a notable presence in T cells (Figure 10A and 10C). Conversely, *S100P* was mainly distributed in NK cells and myeloid cells (Figure 10B). *VEGFC* was not detected in these cells, potentially attributable to the low secretion level of *VEGFC* or the limited amount of data from this single-cell sequencing. Further analysis of T cells showed that *CD244* was mainly distributed in CD8^+^ T cells, with a slightly lower distribution in CD4^+^ T cells (Figure 10D and 10E). Consistent with the results in Figure 10C, *S100P* was only detected in NK cells and myeloid cells, but not in T cells.

**Figure 11.**
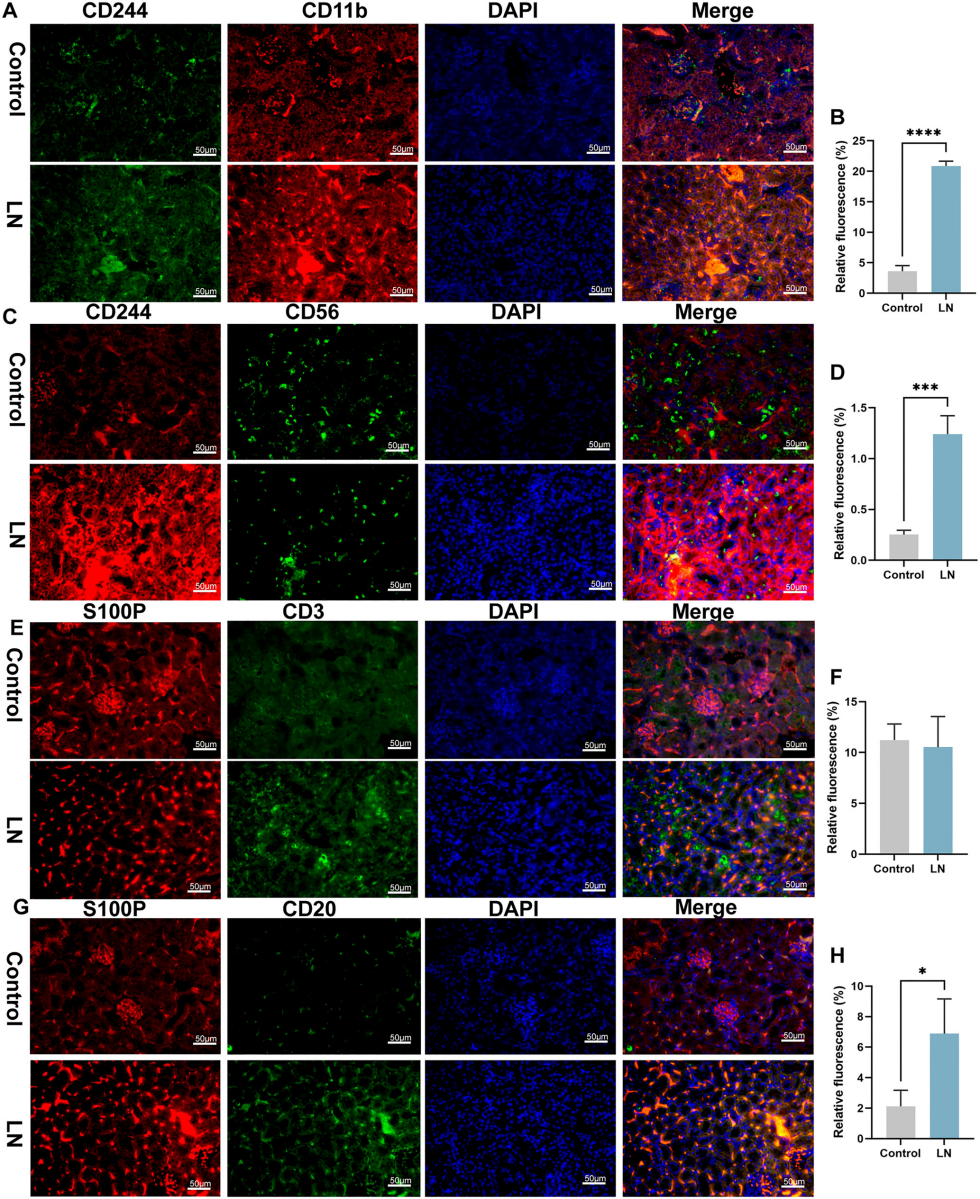
**Immunofluorescence reveals levels of CD244/CD11b, CD244/CD56, S100P/CD3 and S100P/CD20 in the kidney.** (A and B) Relative fluorescence of CD244^+^CD11b^+^; (C and D) Relative fluorescence of CD244^+^CD56^+^; (E and F) Relative fluorescence of S100P^+^CD3^+^; (G and H) Relative fluorescence of S100P^+^CD20^+^. 200 ×, bar ═ 50 µm.*P value < 0.05, **P value < 0.01, ***P value < 0.001. *n* ═ 5 per group. S100P: S100 calcium binding protein P.

**Figure 12.**
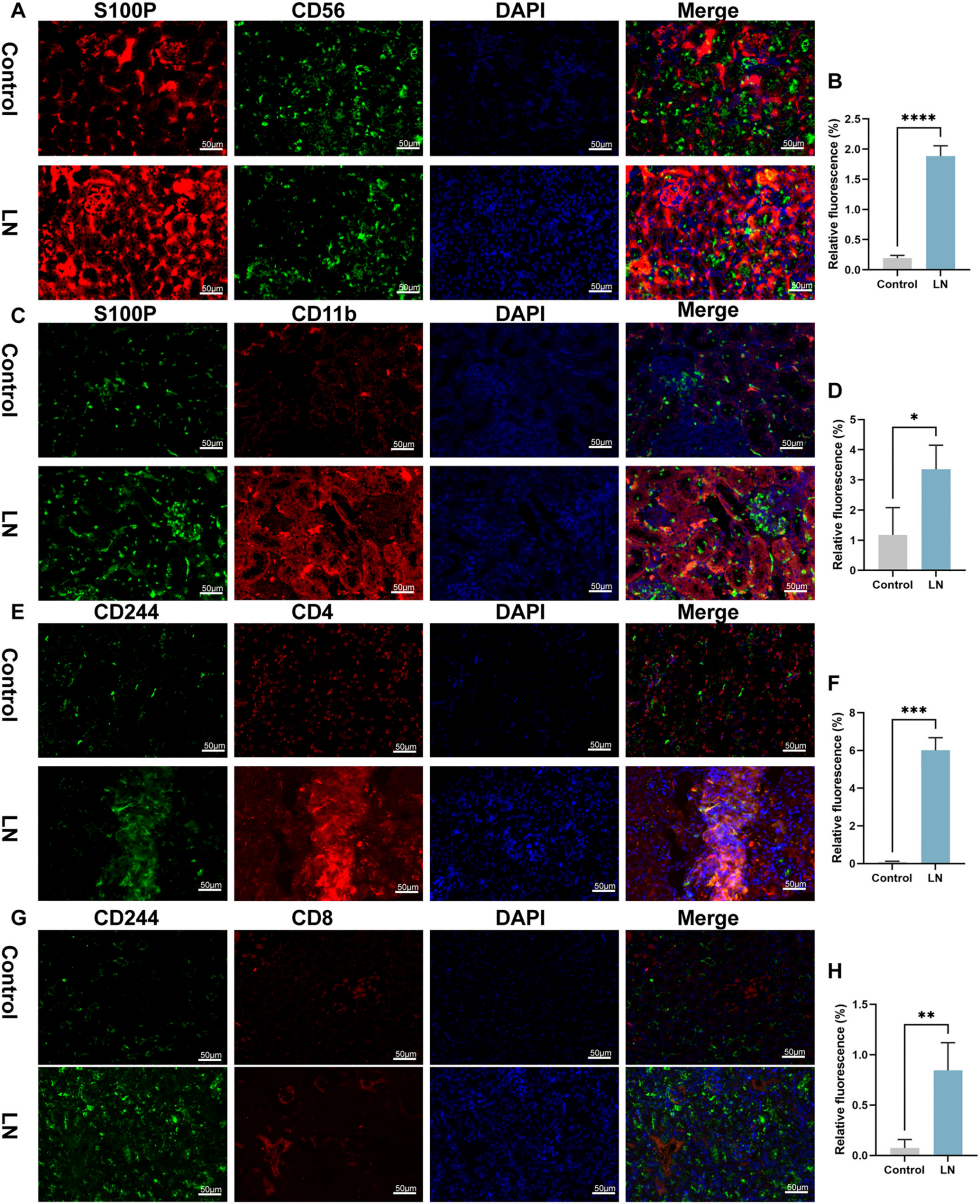
**Immunofluorescence reveals levels of S100P/CD56, S100P/CD11b, CD244/CD4, and CD244/CD8 in the kidney.** (A and B) Relative fluorescence of S100P^+^CD56^+^; (C and D) Relative fluorescence of S100P^+^CD11b^+^; (E and F) Relative fluorescence of CD244^+^CD4^+^; (G and H) Relative fluorescence of CD244^+^CD8^+^. 200 ×, bar ═ 50 µm. *P value < 0.05, **P value < 0.01, ***P value < 0.001, ****P value < 0.001. *n* ═ 5 per group. S100P: S100 calcium binding protein P.

To further verify the distribution of CD244 and S100P in cells, fluorescence staining was performed in mouse kidney (Figures 10–12). Higher CD244 expression was observed in T cells (CD244^+^CD3^+^), B cells (CD244^+^CD20^+^), myeloid cells (CD244^+^CD11b^+^), and NK cells (CD244^+^CD56^+^) in the kidneys of mice in the model group compared to the control group (Figures 10F–10I and 11A–11D, *P* < 0.05). Additionally, S100P expression showed an increase in NK cells (S100P^+^CD56^+^) and myeloid cells (S100P^+^CD11b^+^) (Figure 12A–12D, *P* < 0.05), but not in T cells (S100P^+^CD3^+^) and B cells (S100P^+^CD20^+^) in the model group (Figure 11E–11H). These findings were consistent with the results of single-cell sequencing analysis. Given that CD244 expression was identified in both CD4^+^ T and CD8^+^ T cells in the single-cell sequencing results, analysis of CD244 expression in CD4 T cells (CD244^+^CD4^+^) and CD8 cells (CD244^+^CD8^+^) was conducted in mouse kidneys. As shown in Figure 12E–12H, CD244 expression was found to be increased in the kidneys of mice in the model group compared to the control group. This increase was detected in both CD4^+^ T and CD8^+^ T cells.

## Discussion

LN is currently categorized as an autoimmune disease with a complex progression. It commonly leads to the disruption of B-cell tolerance, the release of autoantibodies, and persistent kidney inflammation [13]. Infection, particularly when hormones and immunosuppressive medications are used, significantly influences the prognosis of LN. These medications weaken humoral and cellular immunity, increasing the likelihood of infections. LN is a leading cause of end-stage renal disease (ESRD) and the most common secondary glomerular disease [20]. Approximately 20% of patients progress to ESRD within a decade of diagnosis, making LN a significant concern in public health. The key focus in the pathogenesis of LN lies in targeting immune pathways due to its unfavorable prognosis [13]. The identified targets in LN are mainly directed against B and T cells, complement activation, signaling pathways, proinflammatory cytokines, and neutrophils [20]. However, these immunosuppressive agents have significant short- and long-term toxicity. Therefore, it is important to search for new targets that can provide safer and more effective options for clinical treatment. An emerging approach to investigate the immunology of LN is continuous screening of new potential therapeutic targets through bioinformatics studies. Bioinformatics is commonly used for screening targets in various illnesses, including oncological diseases. Initially, immune infiltration analysis was used in these diseases [21]. This method is also being used in immune diseases, including LN. However, there is currently limited literature on the analysis of immune infiltration in LN. A previous study analyzed the infiltration of 22 types of immune cells in the kidneys of patients with LN using CIBERSORT. But it excluded the three types of T helper cells (Th1, Th2, and Th17) that play significant roles in LN [8]. A similar problem was observed in a study on SLE bioinformatics [22]. Therefore, it is crucial to clarify the involvement of these T helper cells in order to understand the pathogenesis of LN. In contrast, our study used R software’s ssGESA method to analyze the infiltration of 28 immune cells within the samples and their correlation with the screened IRG-DEGs. Importantly, unlike the previous study, we also established an animal model to verify the reliability of the bioinformatic analysis in vivo [23].

In this study, we included gene expression profiling samples from 20 LN patients and 30 healthy individuals. Using the LIMMA package, we identified 2810 differential genes between LN and healthy individuals. The GO enrichment analysis showed that the differential genes were mainly enriched in a variety of biological processes, cellular components, and molecular functions. Notably, the type I interferon signaling pathway, nucleoplasmic, and protein binding were particularly enriched, highlighting the significance of Th cells. Additionally, the KEGG enrichment analysis showed that the DEGs were mainly enriched in the immune system, which mainly includes the differentiation of Th cell pathways. Th differentiation, including Th1, Th2, and Th17 differentiation, has been shown to play a crucial role in the development of LN disease [24]. There is currently an ongoing debate regarding the dominance of Th1/Th2, with evidence supporting both perspectives. Th1 dominance is linked to IFN-γ production, leading to macrophage activation and exacerbation of the inflammatory response [25]. In contrast, in individuals with SLE, the entry of Th2 lymphocytes suppresses the secretion of Th1 lymphocytes, substantiating the theory of a prevailing “Th2-mediated” immune response [11]. Furthermore, Th17 cells, which produce IL-17, are also believed to play a central role in the progression of T cell-mediated disease [26]. We conducted a single-sample genomic enrichment analysis to assess the distribution of 28 immune cell types in the kidneys of healthy individuals and LN patients. The results revealed increased infiltration of effector memory CD4^+^ T cells, follicular helper T cells, Th1 cells, and Th2 cells in LN patients [27].

Twenty DE-IRGs were then screened using RFA in the kidneys of LN patients to establish a prognostic model of LN. In our study, we used the MRL/*lpr* spontaneous LN mouse model and C57BL/6J mice as controls. Total mRNA from mouse kidneys was extracted, and the analysis revealed a notable increase in CD244 and S100P levels in the LN group (*P* < 0.05), whereas VEGFC levels were significantly decreased (*P* < 0.05). Subsequently, to confirm the expression of CD244, S100P, and VEGFC in the kidneys, we conducted western blotting and immunofluorescence experiments.

CD244, also known as SLAMF4, is a transmembrane protein found in various immune cells, including NK cells, T cells, and other immune cell types. It is an important receptor on the cell surface belonging to the signaling lymphocyte activation molecule (SLAM) family, playing a role in immune regulation [28]. Engaging in delivering either stimulatory or inhibitory signals, CD244 actively controls numerous immune reactions occurring in NK cells, CD8^+^ T cells, and other immune cell populations [29]. In the context of autoimmune diseases, CD244/CD48 co-stimulatory signals are primarily involved in regulating immune cell functions and influencing episodes of autoimmune disease. In SLE, the cell surface protein SLAMF4 is downregulated, leading to a suppression of CD8^+^ T cells’ ability to respond to antigenic stimuli [30]. In patients with active tuberculosis, the cross-linking activation of the CD244/2B4 signaling pathway inhibits the production of IFN-γ, possibly due to its inhibitory effect on Mycobacterium tuberculosis antigen-specific CD4^+^ T cell function [31]. These findings suggest that CD244 may contribute to lupus-associated autoimmunity [32]. Previous studies have mainly focused on examining CD244 expression in CD8^+^ T cells. Nevertheless, our analysis of single-cell sequencing data has revealed a significant expression of CD244 in CD4^+^ T cells as well. Considering the markedly higher expression of CD244 in the kidneys of the LN group in comparison to the control group, it is worth exploring the potential involvement of CD244+CD4+ T cells in the development of LN. Nevertheless, more research is necessary to determine if targeting CD244 could be an effective approach for treating LN.

S100P belongs to the group of proteins called S100, which play a role in controlling cellular processes such as cell cycle progression and differentiation. While initially discovered in the placenta, it has been found to be widely expressed in various internal organs [33]. In certain in vitro studies, IL-11 has been shown to interact with S100P to induce T-cell and macrophage polarization under physiologically relevant conditions [34]. However, the exact mechanisms of action for these two proteins in vivo are currently unknown. This study suggests that in LN, *S100P* and *IL-11* may collaborate to exacerbate inflammation by promoting the differentiation of CD4+ T cells into Th17 cells [35].

Vascular endothelial growth factor (VEGF) is an important growth factor that plays a role in angiogenesis. It has various effects on endothelial cells, including promoting cell growth, preventing cell death, increasing vascular permeability, and promoting cell migration. VEGFC, a subtype of VEGF, plays a role in these processes [36]. Research has revealed that VEGFC participates in the development and maintenance of lymphatic vessels by activating VEGFR3 [37]. Additionally, it has been shown to stimulate the migration of macrophages and lymphocytes in the cancer microenvironment. A recent study using mouse models has revealed that VEGFC activates the CCL21/CCR7 signaling pathway, which promotes the activation and recruitment of naïve T cells to the tumor [38]. In autoimmune diseases, VEGFC has shown a positive correlation with inflammatory cytokines, indicating a potential role in promoting inflammatory activation in autoimmune or autoinflammatory diseases [39]. These findings suggest that VEGFC may also be involved in similar processes in LN.

Previous studies have identified a range of prognostic markers for LN, including traditional biomarkers, such as proteinuria and serum C3, as well as emerging markers like serum primary ANCA-associated vasculitis (ANCA), urinary ALCAM, and indicators from renal biopsies, such as arteriolar C4d deposition, interstitial fibrosis, and tubular atrophy (IFTA) [40]. However, novel biomarkers based on renal tissues are proving to be promising prognostic tools that may have an important role in the long-term prognosis of long-term LN disease. It is reasonable to assume that CD244, S100P, and VEGFC play an important role in the prognosis of LN and are expected to be prognostic. The next step will be to conduct studies with sufficient clinical samples to clarify the clinical evidence for these biomarkers in LN patients. Notably, all three of these biomarkers are specific to the kidneys. Given the complexity of LN, considering all three markers together may better capture the full range of its characteristics. However, it is important to note that these biomarkers can only be assessed through renal biopsy, which is more invasive and potentially damaging than conventional biomarkers.

Our research investigated the correlation between immune cell expression and DE-IRGs in LN. The findings revealed a robust connection between immune cells and S100P, CD244, as well as VEGFC. These findings suggest that CD244, S100P, and VEGFC could potentially be used as biomarkers for LN, either individually or in combination. The current study could serve as a pilot for a further validation study. Nevertheless, it is crucial to acknowledge the restrictions of our investigation. To begin with, our study was conducted retrospectively, which means that it did not offer novel clinical insights. Additionally, the sample size was relatively small. A larger, prospective study is necessary to validate these biomarkers and the infiltration of immune cells in LN.

## Conclusion

*S100P*, *VEGFC*, and *CD244* may serve as novel molecular markers for LN, suggesting potential benefits in its treatment. Furthermore, the role of Th1, Th2, and follicular helper T cells, along with CD4+ T cells, in the progression of LN has been recognized. By targeting these immune cells and biomarkers, immunotherapy offers a promising avenue for LN patients.

## Data Availability

The datasets generated during the current study are available in the GEO repository.

## References

[1] Anders H-J, Saxena R, Zhao M-H, Parodis I, Salmon JE, Mohan C (2020). Lupus nephritis. Nat Rev Dis Primers.

[2] Alforaih N, Whittall-Garcia L, Touma Z (2022). A review of lupus nephritis. J Appl Lab Med.

[3] Bayry J, Bing P-F, Xia W, Wang L, Zhang Y-H, Lei S-F (2016). Common marker genes identified from various sample types for systemic lupus erythematosus. Plos One.

[4] Sezin T, Vorobyev A, Sadik CD, Zillikens D, Gupta Y, Ludwig RJ (2017). Gene expression analysis reveals novel shared gene signatures and candidate molecular mechanisms between pemphigus and systemic lupus erythematosus in CD4(+) T cells. Front Immunol.

[5] Zhong Y, Zhang W, Hong X, Zeng Z, Chen Y, Liao S (2022). Screening biomarkers for systemic lupus erythematosus based on machine learning and exploring their expression correlations with the ratios of various immune cells. Front Immunol.

[6] Sun L, Wang X, Saredy J, Yuan Z, Yang X, Wang H (2020). Innate-adaptive immunity interplay and redox regulation in immune response. Redox Biol.

[7] Frangou E, Georgakis S, Bertsias G (2020). Update on the cellular and molecular aspects of lupus nephritis. Clin Immunol.

[8] Cao Y, Tang W, Tang W (2019). Immune cell infiltration characteristics and related core genes in lupus nephritis: results from bioinformatic analysis. BMC Immunol.

[9] Linke A, Tiegs G, Neumann K (2022). Pathogenic T-cell responses in immune-mediated glomerulonephritis. Cells.

[10] Yang W, Chen X, Hu H (2020). CD4+ T-Cell differentiation in vitro. T-Cell receptor signaling. Methods Mol Biol.

[11] Xiang S, Zhang J, Zhang M, Qian S, Wang R, Wang Y (2022). Imbalance of helper T cell type 1, helper T cell type 2 and associated cytokines in patients with systemic lupus erythematosus: a meta-analysis. Front Pharmacol.

[12] Koga T, Ichinose K, Tsokos GC (2017). T cells and IL-17 in lupus nephritis. Clin Immunol.

[13] Dall’Era M (2017). Treatment of lupus nephritis: current paradigms and emerging strategies. Current Opin Rheumatol.

[14] Cheng C, Zhu R, Liu M, Yang H, Guo F, Du Q (2023). Kunxian capsule alleviates renal damage by inhibiting the JAK1/STAT1 pathway in lupus nephritis. J Ethnopharmacol.

[15] Zhang X, Wang G, Bi Y, Jiang Z, Wang X (2022). Inhibition of glutaminolysis ameliorates lupus by regulating T and B cell subsets and downregulating the mTOR/P70S6K/4EBP1 and NLRP3/caspase-1/IL-1beta pathways in MRL/lpr mice. Int Immunopharmacol.

[16] Nie X, Deng R, Xiang L, Jiang P, Xue Q (2016). Reno-protective effect and mechanism study of Huang Lian Jie Du Decoction on lupus nephritis MRL/lpr mice. BMC Complement Alternat Med.

[17] Su B, Ye H, You X, Ni H, Chen X, Li L (2018). Icariin alleviates murine lupus nephritis via inhibiting NF-kappaB activation pathway and NLRP3 inflammasome. Life Sci.

[18] Liu J, Ma Q, Sun Q, Luo Q, Wang Y, Wang C (2022). Investigating the mechanisms of Jieduquyuziyin prescription improves lupus nephritis and Fibrosis via FXR in MRL/lpr Mice. Oxidat Med Cell Longev.

[19] Xia Y, Jiang C, Yang M, Liu T, Zou X, Li C (2022). SB431542 alleviates lupus nephritis by regulating B cells and inhibiting the TLR9/TGFbeta1/PDGFB signaling. J Autoimmun.

[20] Yung S, Yap DY, Chan TM (2020). A review of advances in the understanding of lupus nephritis pathogenesis as a basis for emerging therapies. F1000Res.

[21] Holtsträter C, Schrörs B, Bukur T, Löwer M.

[22] Zhao X, Zhang L, Wang J, Zhang M, Song Z, Ni B (2021). Identification of key biomarkers and immune infiltration in systemic lupus erythematosus by integrated bioinformatics analysis. J Transl Med.

[23] Wang L, Yang Z, Yu H, Lin W, Wu R, Yang H (2022). Predicting diagnostic gene expression profiles associated with immune infiltration in patients with lupus nephritis. Front Immunol.

[24] Fakhfakh R, Zian Z, Elloumi N, Abida O, Bouallegui E, Houssaini H (2022). Th17 and Th1 cells in systemic lupus erythematosus with focus on lupus nephritis. Immunol Res.

[25] Chen W, Li W, Zhang Z, Tang X, Wu S, Yao G (2020). Lipocalin-2 exacerbates lupus nephritis by promoting Th1 cell differentiation. J Amer Soc Nephrol.

[26] Zhou X, Chen H, Wei F, Zhao Q, Su Q, Lei Y (2019). The inhibitory effects of pentacyclic triterpenes from loquat leaf against Th17 differentiation. Immunol Invest.

[27] Guimarães PM, Scavuzzi BM, Stadtlober NP, Franchi Santos L, Lozovoy MAB, Iriyoda TMV (2017). Cytokines in systemic lupus erythematosus: far beyond Th1/Th2 dualism lupus: cytokine profiles. Immunol Cell Biol.

[28] Mardomi A, Mohammadi N, Khosroshahi HT, Abediankenari S (2020). An update on potentials and promises of T cell co-signaling molecules in transplantation. J Cell Physiol.

[29] Sun L, Gang X, Li Z, Zhao X, Zhou T, Zhang S (2021). Advances in understanding the roles of CD244 (SLAMF4) in immune regulation and associated diseases. Front Immunol.

[30] Kis-Toth K, Comte D, Karampetsou MP, Kyttaris VC, Kannan L, Terhorst C (2015). Selective loss of signaling lymphocytic activation molecule family member 4–positive CD8+ T cells contributes to the decreased cytotoxic cell activity in systemic lupus erythematosus. Arthritis Rheumatol.

[31] Thomas PG, Yang B, Wang X, Jiang J, Cheng X (2013). Involvement of CD244 in regulating CD4+ T cell immunity in patients with active tuberculosis. PLoS One.

[32] Brown DR, Calpe S, Keszei M, Wang N, McArdel S, Terhorst C (2011). Cutting edge: an NK cell-independent role for Slamf4 in controlling humoral autoimmunity. J Immunol.

[33] Truong LD, Shen SS (2011). Immunohistochemical diagnosis of renal neoplasms. Arch Pathol Lab Med.

[34] Kazakov AS, Sokolov AS, Rastrygina VA, Solovyev VV, Ismailov RG, Mikhailov RV (2015). High-affinity interaction between interleukin-11 and S100P protein. Biochem Biophys Res Commun.

[35] Zhang X, Tao Y, Chopra M, Dujmovic-Basuroski I, Jin J, Tang Y (2015). IL-11 Induces Th17 cell responses in patients with early relapsing-remitting multiple sclerosis. J Immunol.

[36] Melincovici CS, Boşca AB, Şuşman S, Mărginean M, Mihu C, Istrate M (2018). Vascular endothelial growth factor (VEGF)—key factor in normal and pathological angiogenesis. Romanian J Morphol Embryol [Internet].

[37] Gao C, Zhu J, Qin Q, Yang X, Jiang Y, Zhang J (2022). The relationship between VEGFC gene polymorphisms and autoimmune thyroiditis. BioMed Res Int.

[38] Deng H, Zhang J, Wu F, Wei F, Han W, Xu X (2023). Current status of lymphangiogenesis: Molecular mechanism, immune tolerance, and application prospect. Cancers.

[39] Chen X, Hu QY, Wang M, Jia J, Teng J, Sun Y (2022). Serum VEGF-C as an evaluation marker of disease activity in adult-onset Still’s disease. Rheumatol Int.

[40] Palazzo L, Lindblom J, Mohan C, Parodis I (2022). Current insights on biomarkers in lupus nephritis: a systematic review of the literature. J Clin Med.

